# Supplementary material for: Membrane type 1 matrix metalloproteinase promotes LDL receptor shedding and accelerates the development of atherosclerosis
Source: Nat Commun. 2021 Mar 25;12:1889. doi: 10.1038/s41467-021-22167-3 (PMC7994674; doi:10.1038/s41467-021-22167-3)
Supplement: Supplementary file 1 — Supplementary Information [file 41467_2021_22167_MOESM1_ESM.pdf]

Online Data Supplements

Supplementary Table 1 Primers

1) Genotyping

   Primers for genotyping MT1-MMP^{Flox} mice

   Forward1-CCTACCATGGGCATAACCTG;

   Reverse-AGGGTGCAGACAGATGGAAG;

   Forward 2-TCTGGATTCATCGACTGTGG.

   Primers for detecting MT1-MMP^{LKO} mice

   Forward1-CCTACCATGGGCATAACCTG;

   Reverse-AGGGTGCAGACAGATGGAAG;

   Forward 2-TCTGGATTCATCGACTGTGG.

2) Site-Directed Mutagenesis and PCR Primers

   E240A: Forward-5'-GGTGGCTGTGCACGCGCTGGGCCATGCCC-3'; Reverse-5'-GGGCATGGCCCAGCGCGTGCACAGCCACC-3'

   A37P: Forward-5'-AGCTTCAGCCCCGAACCCTGGCTACAGCAA-3'; Reverse-5'-TTGCTGTAGCCAGGGTTCGGGGCTGAAGCT-3'

   PCR: MT1-MMP NotI-Forward-5'-TCTAGAGCGGCCGCATGTCTCCCGCCCCAAGACCCCCCCGTTGTC-3'; MT1-MMP HindIII-Reverse-5'-CCATGGAAGCTTTCAGACCTTGTCCAGCAGGGAACGCTGGCAG-3'

   Cre NotI-Forward-5'-TAG CTT GCG GCC GCA TGT CCA ATT TAC TGA CCG TAC ACC AAA ATT TGC-3'; Cre HindIII-Reverse-5'- CCA TGG AAG CTT AGC TAG CTT TGT CGT CAT CAT CCT TAT AGT CCT TA-3'

3) DsiRNA

Scrambled DsiRNA Forward-AUUAGUGUGCGAUGUACCCAGGAAC; Reverse-GUUCCUGGGUACAUCGCACACUAAUAU

MT1-MMP DsiRNA1 Forward-UCCGUGGAAACAAGUACUACCGUTT; Reverse-AAACGGUAGUACUUGUUUCCACGGAAG

MT1-MMP DsiRNA2 Forward- CGCCGACUAAGCAGAAGAAAGAUCA; Reverse-UGAUCUUUCUUCUGCUUAGUCGGCGAA

4) qRT-PCR Primers

Human

GAPDH Forward-GGTGTGAACCATGAGAAGTATGA; Reverse-GAGTCCTTCCACGATACCAAAG

MT1-MMP Froward-TGCCTACCGACAAGATTGATG; Reverse-ATCCCTTCCCAGACTTTGATG

MT2-MMP Froward-ACAACTATCCCATGCCCATC; Reverse-CTTCTCGAAAGAGCCAGTAGC

PCSK9 Forward-CACAGAGTGGGACATCACAG; Reverse-TTTGGCAGAGAAGTGGATCAG

LDLR Forward-TTCACTCCATCTCAAGCATCG; Reverse-ACTGAAAATGGCTTCGTTGATG

IDOL Forward-TGCTGTGTTATGTGACGAGG; Reverse-CTTTGCTACCCGTAAACTGC

SREBP2 Forward-TTCCTGTGCCTCTCCTTTAAC; Reverse-TCATCCAGTCAAACCAGCC

HMGCR Forward-ACAGATACTTGGGAATGCAGAG; Reverse-CTGTCGGCGAATAGATACACC

Mouse

GAPDH Forward-AACTTTGGCATTGTGGAAGG; Reverse-GGATGCAGGGATGATGTTCT

MT1-MMP Froward-TCACCCCAGTCACTCTCAG; Reverse-CTCAGTCCCAAACTTATCCGG

MT2-MMP Froward-CGTTCTAGACAACTACCCCATG; Reverse-TTCTCGGAAAAGCCAGTAGC

MT3-MMP Forward-CGCTACGCATTAACTGGACAG; Reverse-AGGAGTTACATTCTGCCACAC

MT4-MMP Forward-GCTCAAATGCATCGCTTCTG; Reverse-TGTCCTTAAACACCCAGTATCTG

MT5-MMP Forward-AGAAGTGGAGGCAGAAACAC; Reverse-CTCTTCAAAGGTCAGTGGAGTC

MT6-MMP Forward-GTCCCAAAATCCAAATGCCAG; Reverse-AAAATTTCCCCTCGAATGTTGG

ADAM17 Forward-GGGTTTTGCGACATGAATGG; Reverse-GAAAACCAGAACAGACCCAAC

LDLR Froward-ACCCGCCAAGATCAAGAAAG; Reverse-GCTGGAGATAGAGTGGAGTTTG

HMGCR Forward-GCCCTCAGTTCAAATTCACAG; Reverse-TTCCACAAGAGCGTCAAGAG

PCSK9 Forward-TTTTATGACCTCTTCCCTGGC; Reverse-ATTCGCTCCAGGTTCCATG

SREBP2 Forward-CCCTATTCCATTGACTCTGAGC; Reverse CACATAAGAGGATTCGAGAGCG

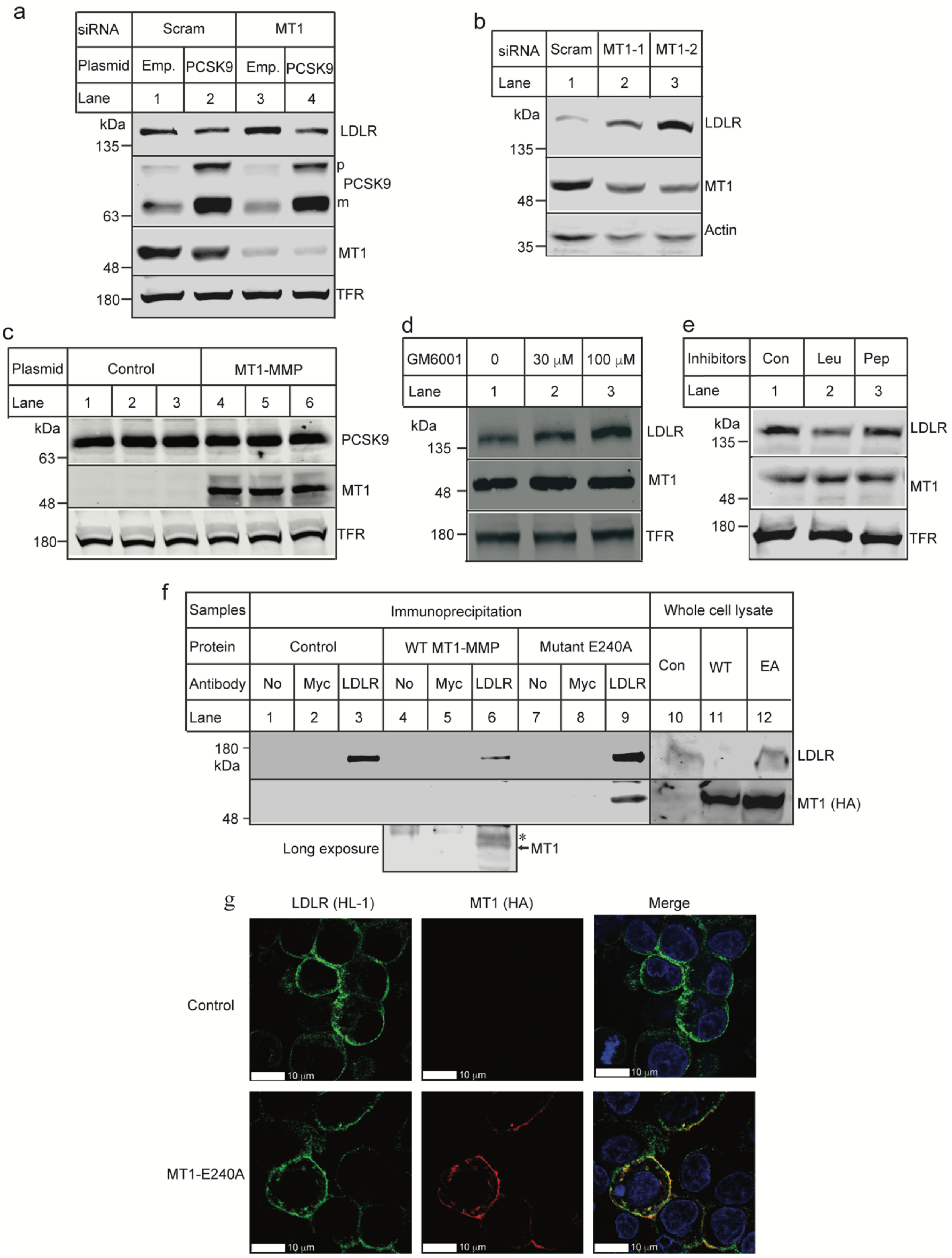
a
siRNA Scram MT1
Plasmid Emp. PCSK9 Emp. PCSK9
Lane 1 2 3 4
kDa
135
63
48
180
LDLR
p
PCSK9
m
MT1
TFR
b
siRNA Scram MT1-1 MT1-2
Lane 1 2 3
kDa
135
48
35
LDLR
MT1
Actin
c
Plasmid Control MT1-MMP
Lane 1 2 3 4 5 6
kDa
63
48
180
PCSK9
MT1
TFR
d
GM6001 0 30 μM 100 μM
Lane 1 2 3
kDa
135
48
180
LDLR
MT1
TFR
e
Inhibitors Con Leu Pep
Lane 1 2 3
kDa
135
48
180
LDLR
MT1
TFR
f
Samples Immunoprecipitation Whole cell lysate
Protein Control WT MT1-MMP Mutant E240A Con WT EA
Antibody No Myc LDLR No Myc LDLR No Myc LDLR
Lane 1 2 3 4 5 6 7 8 9 10 11 12
180
kDa
48
LDLR
MT1 (HA)
Long exposure
*
MT1
g
LDLR (HL-1)
MT1 (HA)
Merge
Control
MT1-E240A
10 μm

Supplementary Fig. 1 Effect of MT1-MMP on LDLR levels. **a** Effect of MT1-MMP on PCSK9-promoted LDLR degradation. Huh7.5 cells were transfected with scrambled (Scram) or MT1-MMP (MT1) siRNA together with either empty plasmid pCDNA3.1 (Emp) or pCDNA3.1 containing PCSK9 cDNA (PCSK9). Whole cell lysate was subjected to Western blot. TFR: transferrin receptor. **b** Effect of MT1-MMP knockdown on LDLR levels. Hepa1c1c7 cells were transfected with scrambled (Scram) or MT1-MMP siRNA, followed by immunoblotting. **c** Effect of MT1-MMP on PCSK9 expression. Huh7.5 cells were transfected with either empty plasmid pCDNA3.1 (Control) or pCDNA3.1 containing MT1-MMP cDNA (MT1-MMP). Whole cell lysate was subjected to Western blot. **d** Effect of GM6001 on LDLR expression. Huh7.5 cells were incubated with GM6001 at concentrations indicated for 16 h. Same amount of whole cell lysate was applied to immunoblotting. **e** Effect of broad spectrum protease inhibitors on LDLR levels. Immunoblotting of same amount of whole cell lysate isolated from Huh7.5 cells treated with Leupeptin (50 μg per ml) or Pepstatin (1 μg per ml) for 5 h. **f** Immunoprecipitation of LDLR. Huh7.5 cells were infected with empty adenovirus (Control), the wild-type, or mutant E204A MT1-MMP adenoviruses. Whole cell lysate was divided equally into three tubes. One tube contained protein G-beads only without antibodies (No), the second tube contained a nonspecific monoclonal antibody (anti-Myc tag) and protein G beads (Myc), and the third tube contained an anti-LDLR monoclonal antibody and protein–G beads (LDLR). The immunoprecipitated proteins and whole cell lysate (Input) were subjected immunoblotting. An anti-HA polyclonal antibody was used to detect HA-tagged MT1-MMP. * non-specific band. **g** Confocal microscopy. HepG2 cells infected with empty adenovirus (Control) or adenovirus containing HA-tagged MT1-E240A were fixed, permeabilization, and then incubated with a mouse anti-LDLR monoclonal and a rabbit anti-HA polyclonal antibody. LDLR, green; MT1-MMP, red; nuclei, blue. Representative images were

showed. Similar results were obtained from three independent experiments (**a to g**). Source data are provided as a Source Data file.

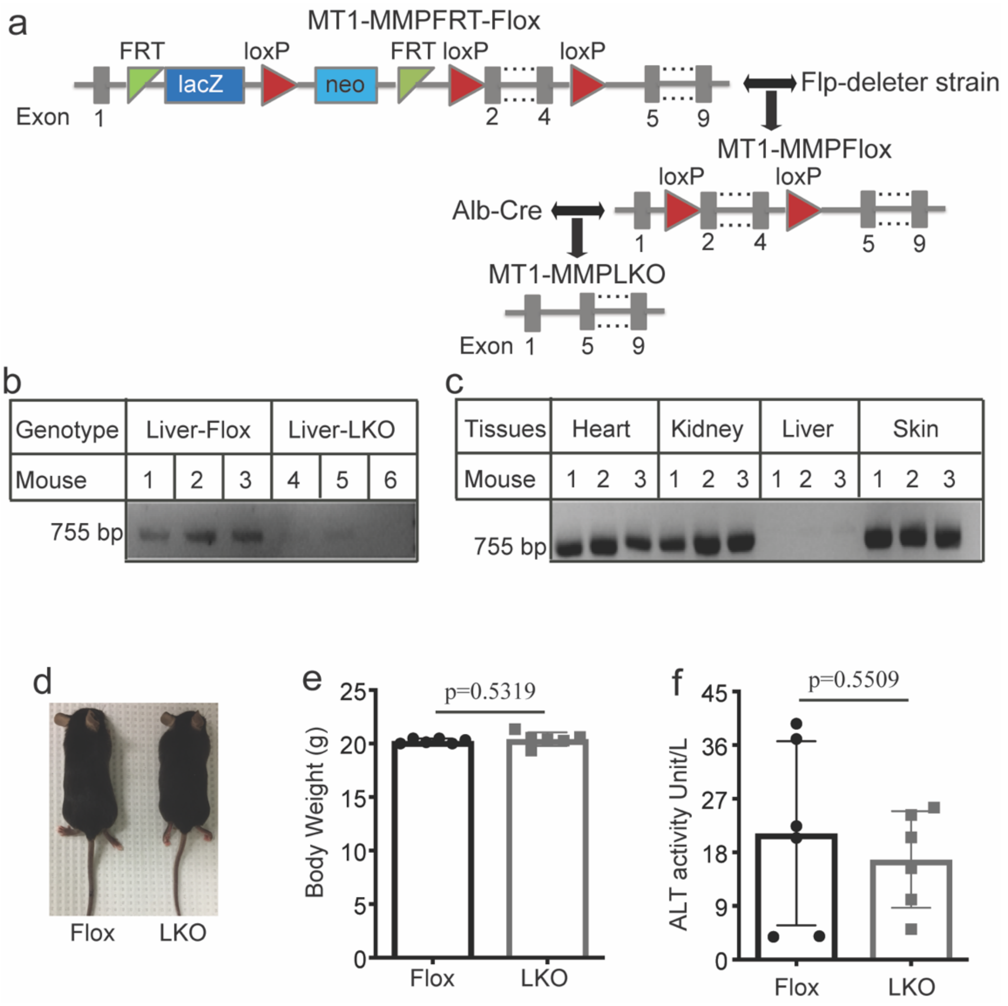

Supplementary Fig. 2 ***MT1-MMP*****^{LKO} mice. a** Schematic of MT1-MMP^{LKO} mice generation. A vector containing the loxP sites that flanked exons 2 to 4 of the *MT1-MMP* gene was used to generate *MT1-MMP*$^{FRT\text{-}Flox}$. *MT1-MMP*$^{FRT\text{-}Flox}$ heterozygotes were crossed to generate homozygotes. The resulting homozygotes were then mated to the Flp-deleter strain (the Jackson laboratory) to obtain *MT1*^{Flox} mice without the FRT-flanked selection marker. *MT1*^{Flox} mice were

then mated with Alb-Cre mice to delete functional MT1-MMP in hepatocytes to generate *MT1*^{LKO} mice. FRT= flippase recognition target. loxP=locus of X-ing of bacteriophage P. **b and c** Genotyping. DNA was extracted from the liver of *MT1*^{Flox} and *MT1*^{LKO} mice **(b)** or different tissues of *MT1*^{LKO} mice **(c)** for genotyping using PCR to detect the LoxP-flanked exons 2–4 of the *MT1-MMP* gene with the AccuStart™ II Mouse Genotyping Kit. One of the two primers was located within of the LoxP sites. A PCR product was only amplified in *MT1*^{Flox} but not in *MT1*^{LKO} mice after recombination leading to deletion of the flanked exons. Representative images were shown. Similar results were obtained from at least three independent experiment. **d** Pictures showing phenotypical similarity in MT1^{Flox} and MT1^{LKO} mice (8 week old). **e** *Body weight of MT1*^{Flox} *and MT1*^{LKO} mice (age of 8 weeks, n=3 males and 3 females per group). **f** Plasma ALT activity measured by a commercial kit. Plasma from each *MT1*^{Flox} or *MT1*^{LKO} mouse (age of 8 weeks, n=3 males and 3 females per group) was subjected to the measurement. Each sample was assayed in triplicate. Student's *t*-test (two-sided) was carried out to determine the significant differences between groups (**e and f**). The significance was defined as $p<0.05$. Values of all data were mean ± SD. Source data are provided as a Source Data file.

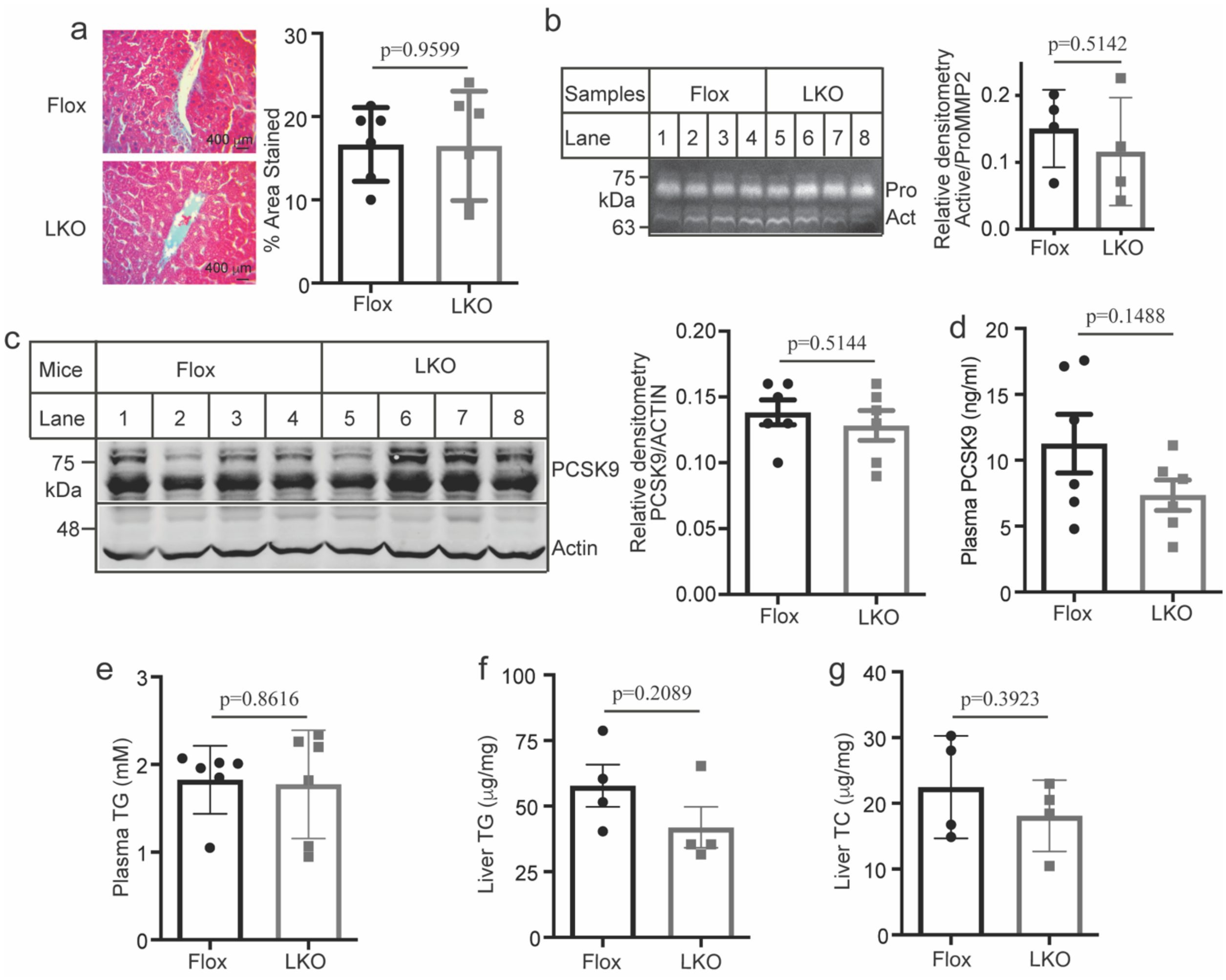

Supplementary Fig. 3 Metabolic effects of *MT1*^{LKO} mice. a Representative Masson's Trichrome staining of liver sections and its quantification (6 Mice per group, age of 8-10 weeks, a regular chow diet). **b** Effect of hepatic knockout of MT1-MMP on MMP2 activation (4 mice per group). Active and pro-MMP2 of plasma samples of mice were detected by the gelatin zymography experiment using 5 μl of plasma from each mouse. The gel was stained with Coomassie Blue R250. The densitometry of active (Act) and pro-MMP2 (Pro) was determined using a Licor Odyssey Infrared Imaging System. The relative densitometry was the ratio of the densitometry of the active form of MMP2 to that of the pro-MMP2 at the same condition. **c** Expression of liver PCSK9 (6 mice per group). Same amount of total proteins in liver homogenate isolated from *MT1*^{Flox} or

MT1^{LKO} mice was subjected to Western Blot. Antibodies used were a rabbit anti-mouse PCSK9 antibody (abcam) and a mouse anti-actin monoclonal antibody. The relative densitometry was the ratio of the densitometry of PCSK9 to that of actin in the same mouse. **d** Plasma levels of PCSK9 (6 mice per group). PCSK9 in same amount of plasma from each mouse was measured using the mouse PCSK9 DuoSet® ELISA kit. **e** Plasma TG (6 mice per group). 5 ul of plasma from each mouse was used to determine plasma TG levels using a commercial kits. **f and g** Levels of TG (**f**) and total cholesterol (TC, **g**) in the liver (4 mice per group). Briefly, lipids were extracted from 4 mg of liver homogenate using the Folch method and then subjected to the determination of TG and TC levels using kits. Representative images were shown. Similar results were obtained from at least three independent experiment (**b and c**). Student's *t*-test (two-sided) was carried out to determine the significant differences between groups (**a to g**). The significance was defined as $p<0.05$. Values of all data were mean ± SD. Source data are provided as a Source Data file.

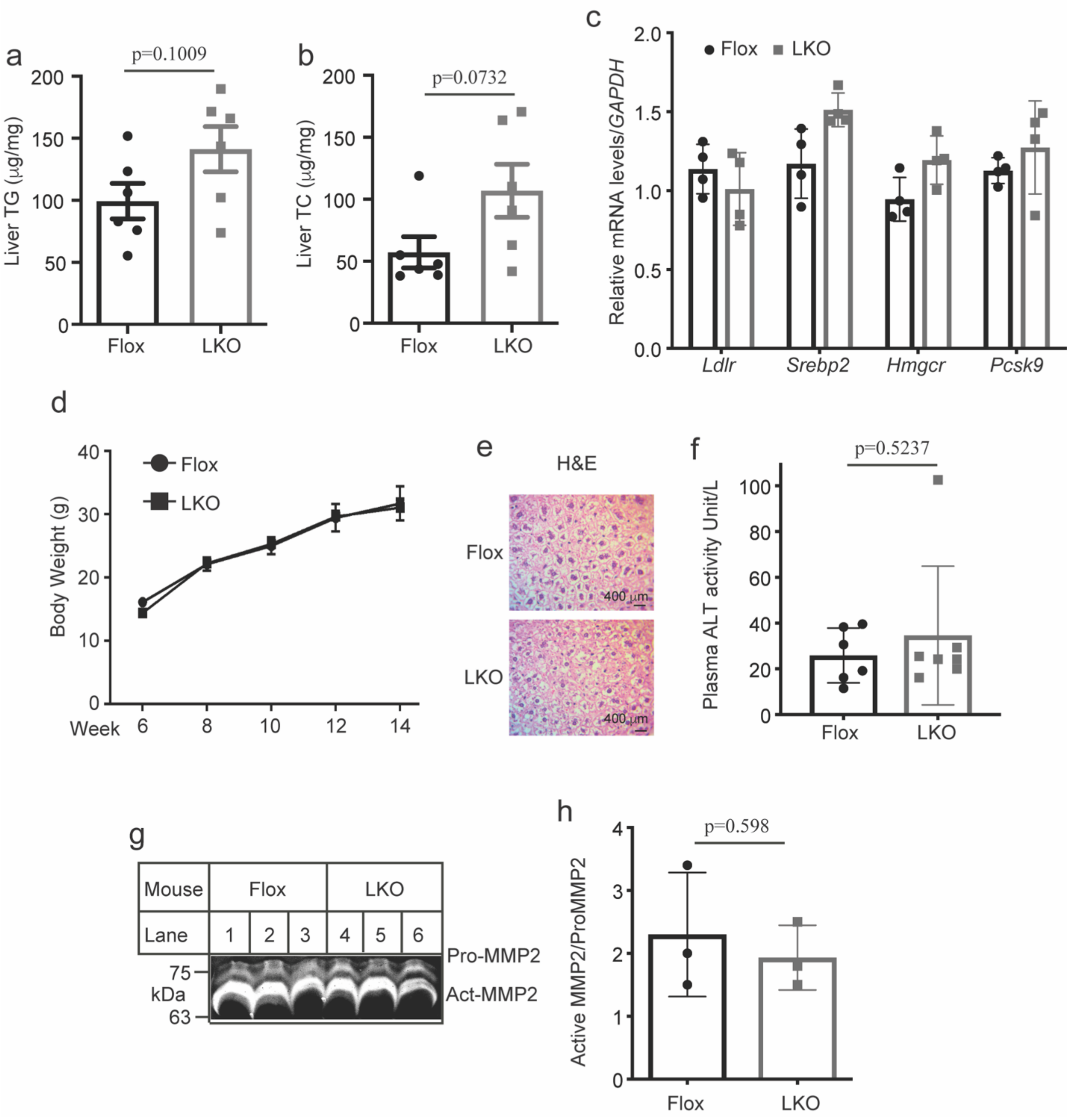

Supplementary Fig. 4 Effects of the Western-type diet. a and b Levels of triglycerides (TG, **a**) and total cholesterol (TC, **b**) in the liver (6 mice per group). Briefly, lipids were extracted from 4 mg of liver homogenate using the Folch method and then subjected to the determination of TG and TC levels using their specific commercial kits. **c** Relative mRNA levels determined by qRT-

PCR (4 mice per group). The relative mRNA levels were the ratio of the mRNA levels of the target genes to that of *GAPDH* at the same condition. **d** Body weight of $MT1^{Flox}$ and $MT1^{LKO}$ mice (4 male mice per group). **e** Liver section staining. Representative figures of H&E staining of cross-sections of liver tissues. Similar results were observed in other mice (5 mice per group). **f** Plasma ALT activity (6 and 7 mice in the Flox and LKO group, respectively). Each sample was assayed in triplicate. **g and h** Gelatin zymography analysis of plasma MMP2 (3 mice per group). The experiment was determined as described in legend to Figure S3b using 5 µl of plasma from each mouse. Representative images were shown. Similar results were obtained from at least three independent experiment (**e and g**). Student's *t*-test (two-sided) was carried out to determine the significant differences between groups (**a, b**, **f, and h**). The significance was defined as $p<0.05$. All data were mean ± SD. Source data are provided as a Source Data file.

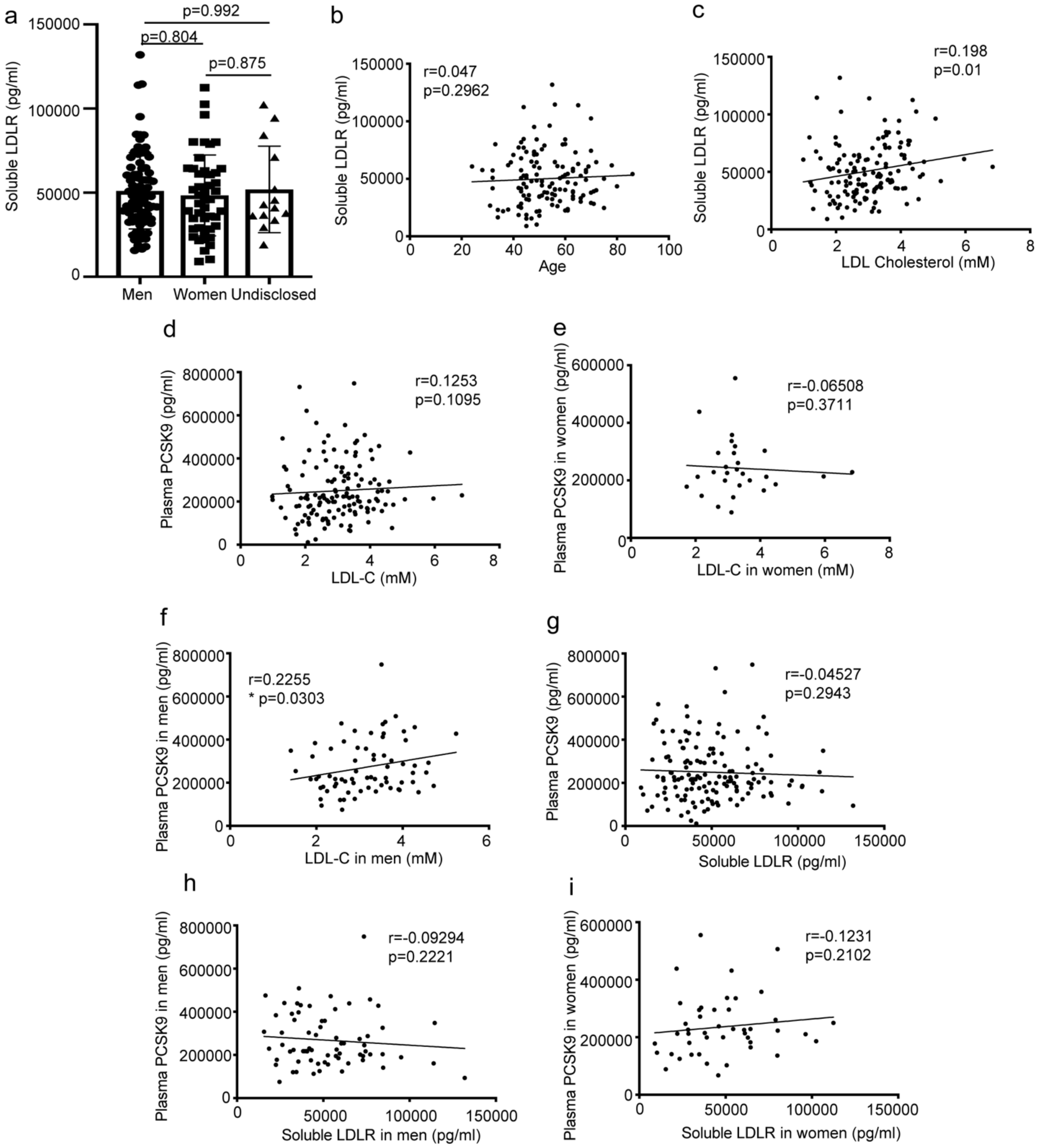

Supplementary Fig. 5 sLDLR in human plasma. a to i Plasma levels of sLDLR and PCSK9. Fasting plasma samples were collected from 148 subjects. sLDLR and PCSK9 was measured using their specific commercial kits. **a** The data were analyzed with one-way Anova and Tukey post-hoc

test using GraphPad Prism 9 (n=87, 46, and 14 individuals in the male, female, and undisclosed group, respectively). Correlation between plasma sLDLR levels and ages **(b)** and LDL-C levels **(c)** or between PCSK9 and cholesterol **(d to f)** or sLDLR **(g to i)** was analyzed with the Pearson's correlation coefficient and plotted using GraphPad Prism 9. Values of all data were mean ± SD. Source data are provided as a Source Data file.

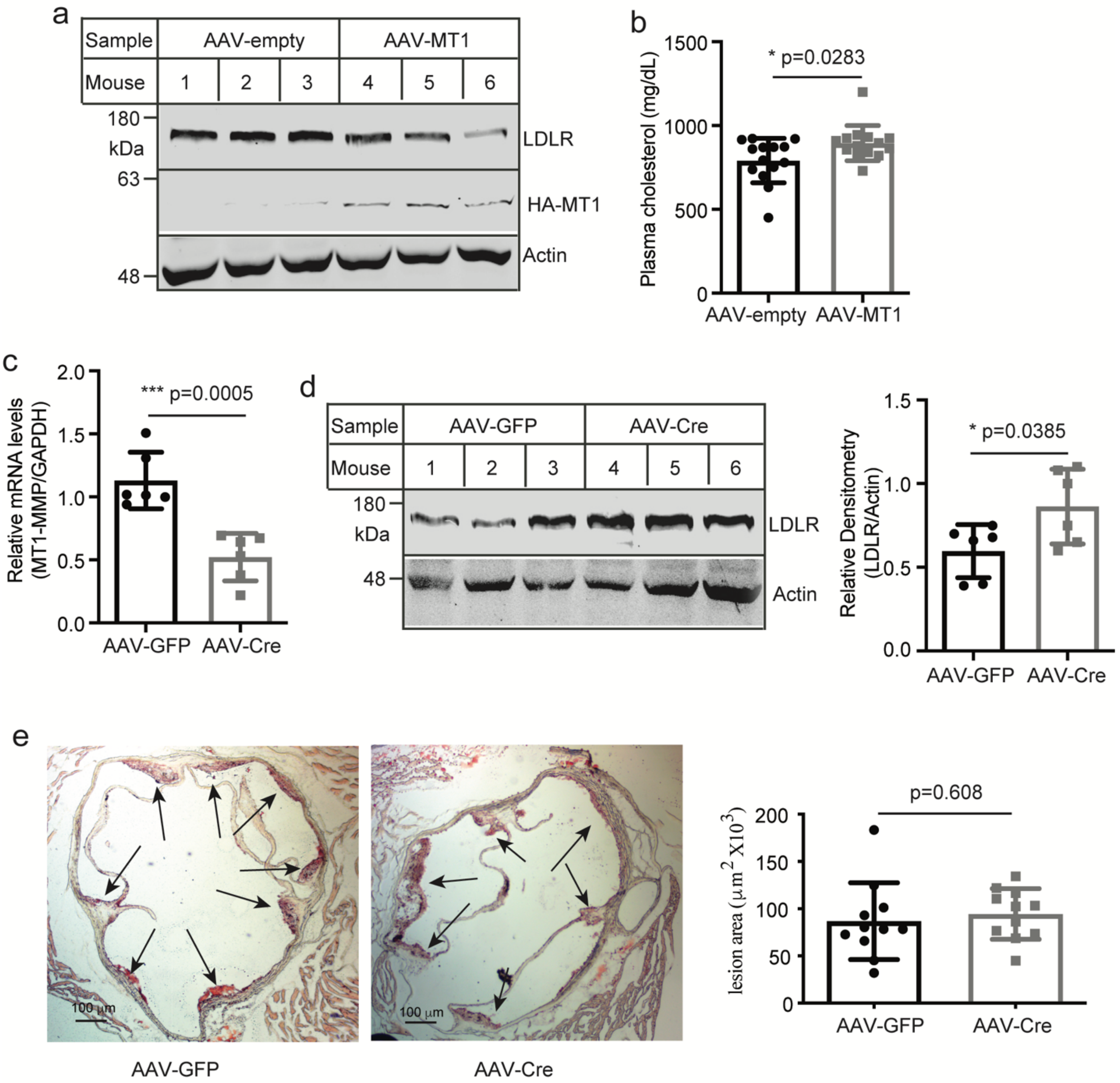

Supplementary Fig. 6 Atherosclerosis study. a and b Overexpression of MT1-MMP. 8-10 week-old male apoE$^{-/-}$ mice were injected with empty AAV or AAV-MT1-MMP (AAV-MT1) and then fed the Western-type diet from TestDiet for 8 weeks. Liver homogenate was subjected to Western blot using a rabbit anti-LDLR polyclonal antibody (3143), a rabbit anti-MT1-MMP

monoclonal antibody and a mouse anti-actin monoclonal antibody representative images were shown. Similar results were obtained from other mice (**a**). 20 μl of plasma from each mouse were used to determine plasma total cholesterol levels using a commercial kits **(b)** (12 mice per group). **c and d** Knockdown of MT1-MMP. 8-10-week male *MT1*^{Flox}/apoE$^{-/-}$ mice were injected with AAV-GFP or AAV-TBG-Cre (AAV-Cre) and then fed the Western diet from Research Diets Inc. for 8 weeks (6 mice per group). Total RNAs were extracted for qRT-PCR (**c**). Liver homogenate was subjected to Western blot using a rabbit anti-LDLR polyclonal antibody (3143) and a mouse anti-actin monoclonal antibody. The relative densitometry was the ratio of the densitometry of LDLR to that of actin in the same mouse (**d**). **e** Atherosclerosis analysis (11 mice per group). 8-10-week-old male *MT1*^{Flox}/apoE$^{-/-}$ mice were injected with AAV-GFP (AAV-GFP) or AAV-TBG-Cre (AAV-Cre) and then fed the Western diet from Research Diets Inc for 8 weeks. The heart was collected and subjected to sectioning. The slides were stained with Oil Red O and imaged on OMAX M837ZL-C140U3 microscope (Magnification 40X). Atherosclerotic lesions in the aortic sinus were quantified using OMAX ToupView. Student's *t*-test (two-sided) was carried out to determine the significant differences between groups. The significance was defined as $p<0.05$. *$p<0.05$, ** $p<0.01$, *** $p<0.001$. Values of all data were mean ± SD. Source data are provided as a Source Data file.
