# Supplementary material for: Membrane type 1 matrix metalloproteinase promotes LDL receptor shedding and accelerates the development of atherosclerosis
Source: Nat Commun. 2021 Mar 25;12:1889. doi: 10.1038/s41467-021-22167-3 (PMC7994674; doi:10.1038/s41467-021-22167-3)
Supplement: Supplementary file 2 — Reporting Summary [file 41467_2021_22167_MOESM2_ESM.pdf]

nature research

Corresponding author(s): Dawei Zhang

Last updated by author(s): Jan 11, 2021

Reporting Summary

Nature Research wishes to improve the reproducibility of the work that we publish. This form provides structure for consistency and transparency in reporting. For further information on Nature Research policies, see Authors & Referees and the Editorial Policy Checklist .

Statistics

For all statistical analyses, confirm that the following items are present in the figure legend, table legend, main text, or Methods section.

n/a	Confirmed	
☐	☒	The exact sample size (n) for each experimental group/condition, given as a discrete number and unit of measurement
☐	☒	A statement on whether measurements were taken from distinct samples or whether the same sample was measured repeatedly
☐	☒	The statistical test(s) used AND whether they are one- or two-sided<br>*Only common tests should be described solely by name; describe more complex techniques in the Methods section.*
☒	☐	A description of all covariates tested
☐	☒	A description of any assumptions or corrections, such as tests of normality and adjustment for multiple comparisons
☐	☒	A full description of the statistical parameters including central tendency (e.g. means) or other basic estimates (e.g. regression coefficient) AND variation (e.g. standard deviation) or associated estimates of uncertainty (e.g. confidence intervals)
☐	☒	For null hypothesis testing, the test statistic (e.g. F, t, r) with confidence intervals, effect sizes, degrees of freedom and P value noted<br>*Give P values as exact values whenever suitable.*
☒	☐	For Bayesian analysis, information on the choice of priors and Markov chain Monte Carlo settings
☒	☐	For hierarchical and complex designs, identification of the appropriate level for tests and full reporting of outcomes
☐	☒	Estimates of effect sizes (e.g. Cohen's d, Pearson's r), indicating how they were calculated

Our web collection on statistics for biologists contains articles on many of the points above.

Software and code

Policy information about availability of computer code

Data collection	Data on atherosclerosis and immunohisochemistry were collected on the OMAX M837ZL-C140U3 microscope. Data on Western blot and qRT-PCR were collected on a Licor Odyssey Infrared Imaging System and StepOnePlus, respectively. Confocal microscopy was performed on a Leica SP5 laser scanning confocal microscope.
Data analysis	GraphPad Prism version 9.0 was used for statistical analysis. Relative stained area was quantified with ImageJ software (1.52S, National institute of Health) using color segmentation and threshold analysis. All image figures were adjusted in Adobe Photoshop 2020 and then made in Adobe Illustrator 2020. The color of most images was discarded in Photoshop. Contrast and exposure were adjusted equally cross the whole image in Photoshop for better visualization. The sizes of images were adjusted in Illustrator.

For manuscripts utilizing custom algorithms or software that are central to the research but not yet described in published literature, software must be made available to editors/reviewers. We strongly encourage code deposition in a community repository (e.g. GitHub). See the Nature Research guidelines for submitting code & software for further information.

Data

Policy information about availability of data

All manuscripts must include a data availability statement. This statement should provide the following information, where applicable:

- Accession codes, unique identifiers, or web links for publicly available datasets
- A list of figures that have associated raw data
- A description of any restrictions on data availability

Source data for all figures are provided within the paper and the Supplementary files. Original data that support this study are available as a Source Data

Field-specific reporting

Please select the one below that is the best fit for your research. If you are not sure, read the appropriate sections before making your selection.

☒ Life sciences ☐ Behavioural & social sciences ☐ Ecological, evolutionary & environmental sciences

For a reference copy of the document with all sections, see nature.com/documents/nr-reporting-summary-flat.pdf

Life sciences study design

All studies must disclose on these points even when the disclosure is negative.

Sample size	Sample-size calculation is not required for the in vitro experiments. All in vitro experiments were repeated at least three times. For animal studies, mice were randomly grouped. Each group contained at least 4 mice to obtain statistical significance while minimizing animal sacrifice based on a publication (Sample Size Calculation in Animal Studies Using Resource Equation Approach, PMID: 29386977, DOI: 10.21315/mjms2017.24.5.11)
Data exclusions	No data exclusion was applied in this study.
Replication	All experiments were repeated at least three times as detailed in Methods and Figure Legends unless stated otherwise. Similar results were obtained at all attempts.
Randomization	Mice were randomly grouped for the atherosclerotic analysis, AAV and adenovirus administration. Human subjects were also randomly recruited. Randomization does not apply to the in vitro cell culture experiments
Blinding	The test of human plasma sLDLR and lipoprotein cholesterol in 148 subjects and quantification of mouse atherosclerotic plaques and oil-red O staining were performed blindly. For cultured cell studies, the same person performed the experiment starting from cell setup, transfection, and sample preparation and analysis. When we loaded samples for Western blot, we could not load them blindly, otherwise, it would be very difficult to present the data. Further, the outcomes of cultured cell experiments were binary, and analysis was performed by software/ equipment uniformly. Thus, blinding was not necessary.

Reporting for specific materials, systems and methods

We require information from authors about some types of materials, experimental systems and methods used in many studies. Here, indicate whether each material, system or method listed is relevant to your study. If you are not sure if a list item applies to your research, read the appropriate section before selecting a response.

Materials & experimental systems

n/a	Involved in the study
☐	☒ Antibodies
☐	☒ Eukaryotic cell lines
☒	☐ Palaeontology
☐	☒ Animals and other organisms
☐	☒ Human research participants
☒	☐ Clinical data

Methods

n/a	Involved in the study
☒	☐ ChIP-seq
☒	☐ Flow cytometry
☒	☐ MRI-based neuroimaging

Antibodies

Antibodies used

772B, a rabbit anti-LDLR antibody. WB, 1:1000 (PMID: 18753623, DOI: 10.1073/pnas.0806312105)
HL-1, a mouse monoclonal anti-LDLR antibody. WB, 1:1000. IP, 1;100. IF, 1:100. (PMID: 18753623, DOI: 10.1073/pnas.0806312105; PMID: 3680245)
3143: a rabbit anti-LDLR polyclonal antibody. WB, 1:5000 (PMID: 18753623, DOI: 10.1073/pnas.0806312105; PMID: 6327078, doi: 10.1016/0092-8674(84)90388-x)
A rat anti-mouse LDLR (Capture antibody from Mouse LDLR DuoSet ELISA). IP, 1:71. R&D Systems. Cat# DY2255. Lot# 326218. https://www.rndsystems.com/products/mouse-ldlr-duoset-elisa_dy2255. It was validated in this study (Fig. 6c). The antibody immunoprecipitated plasma soluble LDLR from the wild type but not LDLR-/- mice.
15A6, a mouse anti-PCSK9 antibody. WB, 1:1000 (PMID: 17080197, doi: 10.1172/JCI29383)
Rabbit anti-mouse PCSK9. WB, 1:500. abcam. Cat# ab185194. Lot# GR3261984-2. https://www.abcam.com/pcsk9-antibody-epr17827-117-ab185194.html
A mouse anti-MT1-MMP monoclonal antibody. IF, 1:100. EMD Millipore, Cat# MAB3329, LOT# 8018903. https://www.emdmillipore.com/CA/en/product/Anti-MMP-14-Antibody-catalytic-domain-clone-LEM-2-63.1,MM_NF-MAB3329
A rabbit anti-MT1-MMP monoclonal antibody. WB, 1:1000. IP, 1:100. Abcam, Cat# ab51074, Lot# GR3213825-2. https://www.abcam.com/mmp14-antibody-ep1264y-ab51074.html
A rabbit anti-MT2-MMP polyclonal antibody. WB, 1:500. ThermoFisher, Cat# PA5-13184, Lot#NL1645034. https://

www.thermofisher.com/antibody/product/MMP15-Antibody-Polyclonal/PA5-13184
A rabbit anti-LRP1 polyclonal antibody. WB, 1:1000. Novus Biologicals, Cat# NBP1-40726, Lot# YG031807C. https://www.novusbio.com/products/lrp-1-antibody-epr3724_nbp1-40726
A mouse anti-calnexin monoclonal antibody. WB, 1:2000. BD Biosciences, Cat#610524, Lot# 80151. https://www.bdbiosciences.com/ca/applications/research/b-cell-research/intracellular-antigens/human/purified-mouse-anti-calnexin-37calnexin/p/610524
A mouse anti-actin monoclonal antibody. WB, 1:5000. BD Biosciences, Cat# 612657, Lot# 11978. https://www.bdbiosciences.com/ca/applications/research/stem-cell-research/mesoderm-markers/human/purified-mouse-anti-actin-ab-5-c4actin/p/612657
A mouse anti-transferrin receptor monoclonal antibody. WB, 1:2000. BD Biosciences, Cat# 612125, Lot# 30217. https://www.bdbiosciences.com/ca/applications/research/stem-cell-research/mesenchymal-stem-cell-markers-bone-marrow/human/positive-markers/purified-mouse-anti-human-transferrin-receptor-2transferrin/p/612125
A goat anti-albumin antibody. WB, 1:5000. ThermoScientific. Cat# PA1-29335. Lot# 22129381D. https://www.thermofisher.com/antibody/product/Human-Serum-Albumin-Antibody-Polyclonal/PA1-29335
Goat anti-apoB antiby. WB, 1:10,000. EMD MIllipore. Cat# AB742. Lot# 3091643. https://www.emdmillipore.com/CA/en/product/Anti-Apolipoprotein-B-Antibody,MM_NF-AB742?ReferrerURL=https%3A%2F%2Fwww.google.com%2F
Rabbit anti-apoE antibody. WB, 1:1000. abcam. Cat# ab183597. Lot# 3217509-1. https://www.abcam.com/apolipoprotein-e-antibody-epr19392-ab183597.html
A rabbit anti-Myc antibody. WB, 1:1000. Upstate (Sigma). Cat# 06-549. Lot# 24165. https://www.sigmaaldrich.com/catalog/product/mm/06549?lang=en&region=CA
Anti-HA EPITOPE TAG (RABBIT) Antibody DyLight™ 800 Conjugated. WB, 1:10,000. Rockland Cat# 600-445-384. https://rockland-inc.com/store/Antibodies-to-More-Epitope-Tags-600-445-384-O4L_12810.aspx
Rabbit anti-HA antibody. WB, 1:1000. ThermoScientific. Cat# OPA1-10980. Lot# NL1645451.https://www.thermofisher.com/antibody/product/HA-Tag-Antibody-Polyclonal/PA1-985
IRDye® 680RD Donkey anti-Rabbit IgG Secondary Antibody. WB, 1:10,000. Li-Cor, Cat# 926-68073. Lot# C11026-02. https://www.licor.com/bio/reagents/irdye-680rd-donkey-anti-rabbit-igg-secondary-antibody
IRDye® 800CW Goat anti-Mouse IgG Secondary Antibody, WB, 1:10,000. Li-Cor. Cat# 926-32210. Lot# C30109-03. https://www.licor.com/bio/reagents/irdye-800cw-goat-anti-mouse-igg-secondary-antibody
IRDye® 680RD Goat anti-Mouse IgG Secondary Antibody, WB, 1:10,000. Li-Cor. Cat# 926-68070. Lot# C11010-01. https://www.licor.com/bio/reagents/irdye-680rd-goat-anti-mouse-igg-secondary-antibody
Goat anti-Mouse IgG (H+L) Cross-Adsorbed Secondary Antibody, Alexa Fluor 568, IF, 1:1000. ThermoFisher. Cat# A11004. Lot# 498389. https://www.thermofisher.com/antibody/product/Goat-anti-Mouse-IgG-H-L-Cross-Adsorbed-Secondary-Antibody-Polyclonal/A-11004
Goat anti-Rabbit IgG (H+L) Cross-Adsorbed Secondary Antibody, Alexa Fluor 488. If, 1:1000. ThermoFisher. Cat# A11008. https://www.thermofisher.com/antibody/product/Goat-anti-Rabbit-IgG-H-L-Cross-Adsorbed-Secondary-Antibody-Polyclonal/A-11008

Validation

All commercial antibodies were validated by the manufacturer as shown in their website. Non-commercial antibodies were validated in publications as listed above.

Eukaryotic cell lines

Policy information about cell lines

Cell line source(s)

HEK293, Hepa1c1c7, McA-RH7777, and HepG2 were originally purchased from ATCC and maintained in the lab. Primary human hepatocytes were provided by Triangle Research Labs. Huh7.5 cells were a kind gift from Dr. N. Kneteman at the University of Alberta (J Hepatol. 2013, 59: 336-43. PMID: 23542347).

Authentication

None of these cell lines was authenticated in the lab.

Mycoplasma contamination

Mycoplasma contamination was tested using DAPI staining, followed by immunofluorescence. All cell lines were tested negative for mycoplasma contamination.

Commonly misidentified lines
(See ICLAC register)

No commonly misidentified lines were used in the study

Animals and other organisms

Policy information about studies involving animals; ARRIVE guidelines recommended for reporting animal research

Laboratory animals

Mice were housed and bred in the animal facility at the University of Alberta. Mice were housed 3 to 5 per cage with free access to H2O in a climate-controlled facility (22 degree, 43% humidity) with a 12 h light/dark cycle. Both male and female MT1Flox and MT1LKO mice (8-12 weeks old) were used in the study. Male apoE-/- mice (8-12 weeks old) were used for the atherosclerosis study.

Wild animals

No wild animals were used in the study.

Field-collected samples

This study did not involve samples collected from field.

Ethics oversight

All animal procedures were approved by the University of Alberta's Animal Care and Use Committee (protocol number AUP00000456) and were conducted in accordance with guidelines of the Canadian Council on Animal Care.

Note that full information on the approval of the study protocol must also be provided in the manuscript.

Human research participants

Policy information about studies involving human research participants

Population characteristics	148 adult Chinese including 87 men (average age=52.6), 46 women (average age=53.7), and 15 individuals, whose gender and age were undisclosed.
Recruitment	Subjects were randomly recruited during their normal physical exam. No self-selection bias or other biases were present during recruitment.
Ethics oversight	The Research Ethic Committee, Institute of Atherosclerosis, Taishan Medical University (Shandong First Medical University)

Note that full information on the approval of the study protocol must also be provided in the manuscript.
